# Comparison of 2.2 mm vs. 2.6 mm corneal incisions in phacoemulsification in hard nuclear cataracts: impact on intraoperative energy and postoperative corneal recovery

**DOI:** 10.3389/fopht.2026.1732396

**Published:** 2026-03-09

**Authors:** Maierdanjiang Ainiwaer, Yingying Hong, Binghe Xiao, Li Ning, Yinghong Ji

**Affiliations:** Eye Institute and Department of Ophthalmology, Eye & ENT Hospital, Fudan University, Shanghai, China

**Keywords:** corneal edema, corneal incision size, hard nuclear cataract, incisional corneal thickness, phacoemalsification

## Abstract

**Purpose:**

This study sought to evaluate the differential impacts of two corneal incision sizes, 2.2 mm and 2.6 mm, on the utilization of intraoperative ultrasound energy and the subsequent postoperative corneal recovery in patients presenting with hard nuclear cataracts.

**Methods:**

A retrospective cohort study was undertaken, encompassing cataract patients classified with nuclear hardness grades III to V according to the Emery-Little classification. Participants were allocated into two groups based on the incision size: 2.2 mm and 2.6 mm. Key metrics, including endothelial cell density (ECD), central corneal thickness (CCT), incisional corneal thickness (ICT), and best-corrected visual acuity (BCVA), were assessed preoperatively and at intervals of 1 day, 1 week, 1 month, and 3 months postoperatively. Additionally, intraoperative phacoemulsification parameters and any complications were documented.

**Results:**

The study encompassed a total of 100 eyes, with 50 eyes in each group. No statistically significant differences were detected in cumulative dissipated energy (CDE) or ultrasound time (UST) between the two groups (P > 0.05). Both groups exhibited a significant postoperative decline in ECD (P < 0.05), yet no significant intergroup difference was observed in the magnitude of this reduction (P > 0.05). Central corneal thickness (CCT) and intraocular pressure (ICT) exhibited a statistically significant increase at both 1 day and 1 week postoperatively when compared to baseline measurements (P < 0.05). Notably, the increase in ICT was significantly more pronounced in the 2.2 mm incision group at both time points (P < 0.05). Best-corrected visual acuity (BCVA) showed significant improvement in both groups following surgery (P < 0.05), with no significant intergroup differences observed at any follow-up interval (P > 0.05). Additionally, no significant difference in central corneal edema was detected between the two groups (P > 0.05).

**Conclusion:**

The corneal incision size (2.2 mm versus 2.6 mm) did not influence the use of intraoperative ultrasound energy or result in differential corneal endothelial cell loss. However, smaller incisions (2.2 mm) were associated with increased short-term edema at the incision site, potentially impacting early wound healing.

## Introduction

Over recent decades, cataract surgery has experienced substantial advancements, enhancing refractive outcomes, safety, and cost-effectiveness. With the progression of phacoemulsification technology, the selection of incision size has emerged as a critical factor affecting surgical outcomes. The shift from extracapsular cataract extraction (ECCE) to small-incision and microincision cataract surgery (MICS) signifies a significant advancement in ophthalmic surgery. ECCE necessitates a substantial limbal incision and suturing, and it continues to be prevalent in developing countries due to its cost-effectiveness (1).

Nonetheless, with the widespread adoption of phacoemulsification and foldable intraocular lenses (IOLs), small-incision and MICS have become the standard practices (2). In contrast to ECCE, small-incision phacoemulsification employs smaller clear corneal incisions, thereby minimizing the requirement for sutures. This approach results in reduced tissue trauma, accelerated wound healing, and diminished risks of leakage and endophthalmitis (3). Furthermore, MICS has the potential to decrease surgically induced astigmatism (SIA) (4), thereby enhancing visual outcomes and shortening recovery periods. However, the reduction of incision size from 2.5–3.2 mm (small-incision) to 1.6–2.2 mm (microincision) is not without its challenges (5). Although the surgical procedures are analogous, smaller incisions may elevate mechanical stress at the wound site due to instrument movement during phacoemulsification (6). Consequently, the optimal incision size remains a topic of debate and necessitates further research.

This retrospective cohort study compared the effects of 2.2 mm and 2.6 mm corneal incisions on intraoperative energy use and postoperative corneal recovery in patients with hard nuclear cataracts.

## Methods

### Study population

This retrospective study encompassed patients who underwent phacoemulsification and IOL implantation at the Eye and ENT Hospital of Fudan University from June 2022 to October 2023. The study received approval from the hospital’s ethics committee and was conducted in accordance with the principles outlined in the Declaration of Helsinki. Informed written consent was obtained from all participants. The inclusion criteria were as follows: (1) patients with age-related cataracts; (2) nuclear hardness graded III to V according to the Emery-Little classification; (3) age range of 50 to 90 years; (4) pupil dilation of at least 7 mm; (5) normal corneal transparency with an endothelial cell density (ECD) of at least 2000 cells/mm^2^; (6) absence of significant fundus pathology; and (7) willingness to participate and comply with follow-up requirements. The exclusion criteria included: (1) presence of ocular or systemic diseases affecting the corneal endothelium; Patients with poorly controlled diabetes (HbA1c >8%) or diabetic retinopathy (any stage) were excluded from the study; (2) glaucoma; (3) uveitis; (4) history of previous ocular surgery or trauma; and (5) severe retinal pathology.

### Preoperative examination

All patients underwent a thorough ophthalmic evaluation, which included assessments of visual acuity, slit-lamp biomicroscopy, intraocular pressure measurement, B-scan ultrasonography, optical coherence tomography (OCT), corneal topography using the Pentacam HR (OCULUS), corneal endothelial cell microscopy with the EM-3000 (Tomey), axial length measurement, and IOL power calculation via the IOL Master 700 (ZEISS). Preoperative best-corrected visual acuity (BCVA), endothelial cell density (ECD), central corneal thickness (CCT), peripheral corneal thickness (PCT), and lens nuclear hardness were documented.

### Surgical technique

All surgical interventions were conducted by a single, highly experienced surgeon employing standardized surgical techniques. Preoperative mydriasis was induced using compound tropicamide eye drops, and topical anesthesia was administered with 0.4% oxybuprocaine hydrochloride. During the procedure, Viscoat (Bausch & Lomb) and balanced salt solution (BSS, Alcon) were utilized. Two incision sizes were employed: 2.2 mm and 2.6 mm clear corneal incisions. Following the injection of viscoelastic, a 5.5 mm continuous curvilinear capsulorhexis was executed, succeeded by hydrodissection. Phacoemulsification was performed utilizing the Centurion system (Alcon) with the parameters detailed in **Table 1**. We used the same phaco tip design (45° Kelman tip, Alcon) in both groups.2.2 mm ABS^®^ Phaco Sleeve (Alcon).

**Table 1:** Intraoperative phacoemulsification parameters.

Parameter	Value/Mode
Phacoemulsification mode	Continuous
Torsional amplitude	0–90%
Longitudinal power	0
Fluidics system	Gravity-fed
Bottle height	100 cm
Vacuum	360 mmHg
Flow rate	30 cm^3^/min
Burst time	–
Off time	–

And 2.6 mm ABS^®^ Phaco Sleeve (Alcon) were used in the two groups. Following IOL implantation, the viscoelastic substance was evacuated, and a dose of 1 mg/0.1 ml cefuroxime was administered into the anterior chamber. The surgical incision was hydrated to promote self-sealing. Postoperative pharmacological management included the administration of 1% prednisolone acetate, 0.5% levofloxacin, and 0.1% pranoprofen eye drops.

### Postoperative follow-up

**Figure 1:**
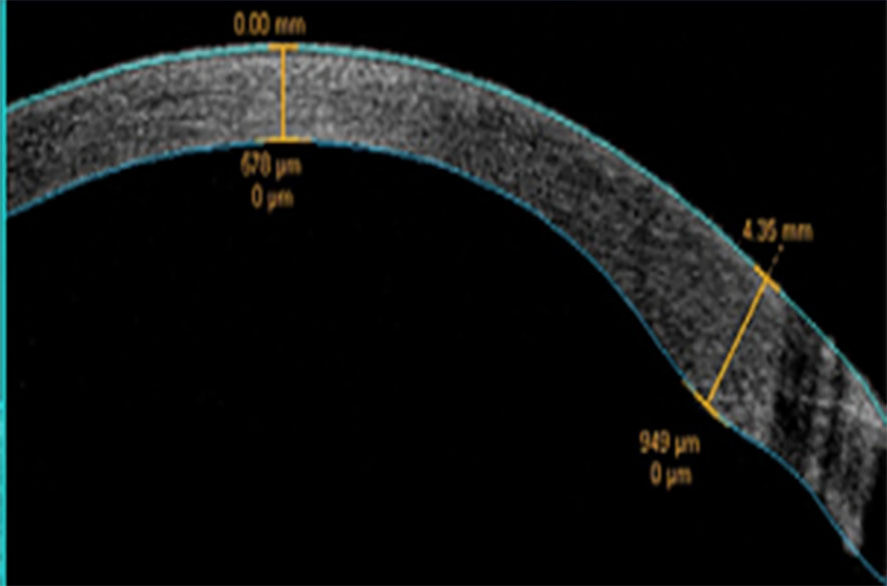
Anterior segment OCT measurement of corneal thickness at the incision site.

Intraoperative UST and CDE were documented by the phacoemulsification system. Postoperative evaluations were scheduled at intervals of 1 day, 1 week, 1 month, and 3 months. These assessments encompassed the measurement of BCVA using the logMAR notation, slit-lamp examination for grading corneal edema (7) and assessing the position of the IOL, corneal thickness measurement via Pentacam HR, and incisional corneal thickness (ICT) evaluation using anterior segment optical coherence tomography (OCT) (CASIA2, Tomey) (**Figure 1**). ECD was measured using specular microscopy (EM-3000, Tomey).

### Statistical analysis

Statistical analyses were performed using SPSS version 25.0. The normality of the data was assessed using the Kolmogorov–Smirnov test, and the appropriate statistical methods were used. Using Mann–Whitney U tests and t tests, disparities in CDE and UST among the two groups were compared, as were postoperative changes in ECD, CCT and ICT. The categorical data are presented as percentages and numbers, and chi-square or continuity-corrected chi-square tests were used to analyze the results. A p value less than 0.05 indicated statistical significance.

## Results

### Baseline characteristics and intraoperative parameters

The study included a total of 100 eyes, with 50 eyes in each group. Preoperatively, no significant differences were observed between the two groups in terms of age, gender, ECD, CCT, PCT), or BCVA) (P > 0.05) (**Table 2**). Similarly, no significant differences were noted in CDE or UST between the groups (P > 0.05) (**Table 3**).

**Table 2:** Preoperative baseline characteristics of the study groups.

Parameter	2.2 mm Group (n=50)	2.6 mm Group (n=50)	*P* value
Age (years)	69.3 ± 9.0	70.2 ± 8.9	0.682
Female, n (%)	23 (46.0)	26 (53.1)	0.247
Preoperative BCVA (logMAR)	0.93 ± 0.39	0.96 ± 0.42	0.462
Preoperative ECD (cells/mm^2^)	2647.6 ± 267.8	2633.3 ± 231.7	0.732
Preoperative CCT (μm)	533.5 ± 36.7	527.3 ± 36.1	0.277
Preoperative PCT (μm)	753.36 ± 46.33	750.37 ± 42.58	0.329

BCVA, best-corrected visual acuity; ECD, endothelial cell density; CCT, central corneal thickness; PCT, peripheral corneal thickness. Data are presented as mean ± standard deviation or number (percentage).

**Table 3:** Cumulative dissipated energy (CDE) and ultrasound time (UST) during surgery.

Parameter	CDE	UST
2.2 mm	14.1 ± 5.1	55.1 ± 25.7
2.6 mm	13.8 ± 8.3	54.3 ± 27.4
*P* value	0.875	0.759

CDE, cumulative dissipated energy; UST, ultrasound time. Data are presented as mean ± standard deviation.

### Postoperative corneal changes

Postoperative Corneal Changes: CCT exhibited a significant increase at both 1 day and 1-week post-surgery in both groups (P < 0.05), returning to baseline levels by 1 month. There were no significant intergroup differences in CCT changes (P > 0.05) (**Table 4**). ECD significantly decreased postoperatively in both groups (P < 0.05), without significant intergroup differences (P > 0.05) (**Table 5**). ICT significantly increased at 1 day and 1 week postoperatively in both groups (P < 0.05), with a significantly greater increase observed in the 2.2 mm group at both time points (P < 0.05) (**Table 6**).

**Table 4:** Postoperative central corneal thickness (CCT) changes.

Parameter	2.2 mm	2.6 mm	*P* value
CCT (μm)			
Preoperative	533.53 ± 36.65	527.34 ± 36.42	0.277
Postoperative 1 day	582.94 ± 42.86#	578.77 ± 37.64#	0.229
Postoperative 1 week	557.12 ± 40.43#	561.32 ± 42.35#	0.363
Postoperative 1 month	536.57 ± 38.52	535.24 ± 39.86	0.265
Postoperative 3 months	532.78 ± 33.90	529.47 ± 31.12	0.351
Change from baseline			
Δ at 1 day	52.10 ± 23.20	51.21 ± 27.09	0.414
Δ at 1 week	25.82 ± 13.17	28.59 ± 19.61	0.367
Δ at 1 month	3.22 ± 5.11	4.32 ± 4.95	0.402
Δ at 3 months	3.31 ± 4.39	3.82 ± 5.46	0.118

#P < 0.05 vs. preoperative value within the same group (Paired t-test); Δ, change.

**Table 5:** Postoperative endothelial cell density (ECD) changes.

Parameter	2.2 mm Group (cells/mm^2^)	2.6 mm Group (cells/mm^2^)	*P* value
ECD			
Preoperative	2647.63 ± 135.98	2633.08 ± 231.70	0.732
Postoperative 1 day	2407.19 ± 285.56#	2399.37 ± 283.07#	0.886
Postoperative 1 week	2335.90 ± 304.60#	2294.56 ± 314.17#	0.507
Postoperative 1 month	2270.67 ± 350.41#	2219.76 ± 340.79#	0.457
Postoperative 3 months	2212.21 ± 400.71#	2197.34 ± 354.36#	0.839
Change from baseline			
Δ at 1 day	-231.40 ± 131.86	-245.23 ± 211.02	0.331
Δ at 1 week	-313.18 ± 174.02	-357.78 ± 267.26	0.146
Δ at 1 month	-399.37 ± 233.67	-415.39 ± 216.52	0.329
Δ at 3 months	-416.10 ± 297.95	-420.25 ± 244.94	0.213

#P < 0.05 vs. preoperative value within the same group (Paired t-test); Δ, change.

**Table 6:** Postoperative changes in incisional corneal thickness (ICT).

Parameter	2.2 mm Group (% change)	2.6 mm Group (% change)	*P* value
ICT (μm)			
Preoperative (μm)	753.36 ± 46.33	750.37 ± 42.58	0.329
%Change from baseline			
At 1 day	26.85 ± 3.16	19.55 ± 2.53	0.021*
At 1 week	20.87 ± 2.39	12.39 ± 0.95	0.037*

Data are presented as mean ± standard deviation. P values for intergroup comparisons were derived from independent t-tests. Statistically significant (P < 0.05).

*: Statistically significant (P < 0.05).

### Visual outcomes and complications

BCVA showed significant improvement in both groups following surgery (P < 0.001), with no significant differences between the groups at any follow-up interval (P > 0.05) (**Table 7**). No intraoperative complications, such as posterior capsule rupture, were reported. Additionally, no postoperative complications, including posterior capsule opacification (PCO) or endophthalmitis, were observed. The extent of central corneal edema did not differ significantly between the groups (P > 0.05) (**Table 8**).

**Table 7:** Postoperative best-corrected visual acuity (BCVA, logMAR).

BCVA (logMAR)	2.2 mm	2.6 mm	*P* Value
Preoperative	0.91 ± 0.49	0.95 ± 0.45	0.380
Postoperative 1 day	0.23 ± 0.18	0.32 ± 0.26	0.396
Postoperative 1 week	0.18 ± 0.09	0.18 ± 0.17	0.652
Postoperative 1 month	0.07 ± 0.07	0.08 ± 0.11	0.912
Postoperative 3 months	0.06 ± 0.05	0.07 ± 0.10	0.950

BCVA, best-corrected visual acuity. Data are presented as mean ± standard deviation. P values were derived from independent t-tests.

**Table 8:** Central corneal edema at postoperative day 1 and week 1.

Parameter	Group	None (0)	Mild (1)	Moderate (2)	Marked (3)	Severe (4)	*P* value
Postoperative 1 day	2.2 mm	40	6	4	0	0	0.498
	2.6 mm	39	7	4	0	0	
Postoperative 1 week	2.2 mm	48	2	0	0	0	0.545
	2.6 mm	45	4	0	0	0	

Grading, 0, None; 1, Mild; 2, Moderate; 3, Marked; 4, Severe. P values were derived from Chi-square tests.

## Discussion

ECCE was the predominant technique prior to the advent of phacoemulsification. Despite not utilizing ultrasound energy, ECCE necessitates a large incision and subsequent suturing, which results in increased corneal trauma (7). With the progression of phacoemulsification technology, small-incision surgery has gained prominence due to its association with expedited recovery and reduced astigmatism (8). Clear corneal incisions present several benefits, including straightforward construction, minimal conjunctival damage, and the ability for sutureless closure (8). Nonetheless, the size of the incision may impact the extent of mechanical and thermal injury incurred during surgery. Although micro- MICS may decrease energy consumption (9), it could also elevate wound stress and prolong healing (10).

Small-incision cataract surgery (SICS) is typically performed through a clear corneal incision ranging from 2.5 to 3.2 mm, whereas micro-incision cataract surgery (MICS) generally utilizes incisions between 1.5 and 2.2 mm, with the potential for even smaller sizes. However, MICS imposes higher demands on phacoemulsification equipment and intraocular lens (IOL) dimensions (11, 12).

Previous studies have shown no significant difference in the safety profile between SICS and MICS (9). Nevertheless, their intraoperative and postoperative efficacy outcomes diverge. Some reports indicate that, aside from differences in intraoperative phacoemulsification parameters, the two techniques yield comparable results in terms of postoperative visual acuity, corneal endothelial cell loss, astigmatism, and other complications (13, 14). The primary distinction lies in the reduced ultrasound energy utilization and shorter phaco time observed with MICS. In contrast, other studies suggest that, beyond intraoperative parameters, MICS facilitates faster recovery of corrected visual acuity (15), potentially leading to improved clinical outcomes. Given the variations in incision sizes and surgical platforms across these studies, further investigation is warranted to optimize surgical strategies for specific patients.

Another study reported that in patients with hard nuclei, a 1.8 mm micro-incision resulted in a higher cumulative dissipated energy (CDE) and ultrasound time (UST) compared to a 2.2 mm micro-incision, potentially leading to greater corneal endothelial cell damage (16). This finding contradicts several previous studies. The discrepancy suggests that an excessively small incision might compress the phacoemulsification sleeve, compromising irrigation flow and anterior chamber stability. This compromised stability could, in turn, slow the dissipation of thermal energy, paradoxically increasing the risk of endothelial injury (17).

These observations highlight that surgical outcomes can vary significantly depending on the patient population (e.g., hard nuclei), the surgical platform, and the specific incision size employed. Therefore, in the present study, we compared a 2.2 mm micro-incision with a 2.6 mm small-incision specifically in patients with hard nuclear cataracts. All other surgical parameters were standardized to minimize confounding variables, allowing us to determine which approach yields superior clinical outcomes in this specific patient cohort.

We compared intraoperative ultrasound time (UST) and cumulative dissipated energy (CDE). The results indicated no significant differences in the mean CDE or UST between the two groups during cataract surgery on hard nuclei. This suggests that utilizing either a 2.2 mm or a 2.6 mm incision does not significantly impact intraoperative phacoemulsification parameters in hard nucleus cases. This finding contrasts with previous comparative studies involving two micro-incision sizes. It is plausible that the more confined space of a 1.8 mm incision may restrict surgical maneuverability, leading to increased CDE and UST—a phenomenon not observed with the 2.2 mm and 2.6 mm incisions used in our study.

During the three-month postoperative follow-up period, endothelial cell density (ECD) exhibited a continuous decline from baseline levels in both groups, with no significant difference observed between them. We note that the corneal endothelial cell loss in our findings are consistent with reports on small-incision phacoemulsification in hard cataracts (18, 19). Central corneal thickness (CCT) increased significantly one day and one week postoperatively compared to preoperative values but returned to baseline levels by one month. Best-corrected visual acuity (BCVA) showed significant improvement on the first postoperative day compared to preoperative levels, again with no significant intergroup difference. The degree of central corneal edema similarly showed no significant difference between the two groups. These findings align with previous research, indicating that the incision size within this range does not markedly influence corneal endothelial loss or central corneal edema.

However, specific measurements at the incision site itself revealed a notable difference. Compared to the preoperative peripheral corneal thickness, the increase in corneal thickness at the incision site on postoperative day 1 and week 1 was significantly smaller in the 2.6 mm incision group than in the 2.2 mm incision group. The observed incision-site edema in the 2.2−mm group was transient and self-limiting, resolving within the first postoperative week without clinical sequelae. Importantly, it did not lead to delayed visual recovery (BCVA was comparable between groups at 1 week and 1 month) and wound instability (no cases of wound leak or Seidel positivity). Previous studies have suggested that micro-incision phacoemulsification might generate greater incision stress compared to small-incision techniques, potentially inducing more pronounced architectural changes at the wound and consequently delaying its healing (10). During maneuvers such as nucleus splitting and fragment rotation, the effective range of motion and force application of the instruments inside the eye are constrained. To manipulate hard nuclei more effectively, surgeons may sometimes unconsciously increase lateral twisting or wiggling of the instrument at the incision site. This can easily lead to microscopic tears, edema, or even Descemet’s membrane detachment in the local tissue around the incision. In contrast, a slightly larger incision may clearance for the instrument, distributing mechanical stress more widely and thus reducing the risk of severe localized damage. The study of Hepokur et al. confirmed that Smaller corneal incisions slightly reduced SIA, but tended to induce more endothelial cell loss, comparing 2.2 mm and 2.8 mm corneal incision sizes (20). Our results are consistent with this hypothesis, smaller incisions tend to cause more or faster jet streams and might not cool the phaco tip efficiently compared to larger incisions.

In this study, we observed no significant differences in CDE, UST, ECD loss, or central corneal edema between incisions measuring 2.2 mm and 2.6 mm. However, the 2.2 mm incision group exhibited greater short-term thickening at the incision site, indicative of increased wound stress and more endothelial cell loss around the incision site.

## Conclusion

In cases involving patients with hard nuclear cataracts, there was no discernible difference in intraoperative energy usage or overall corneal endothelial damage between the 2.2 mm and 2.6 mm incisions. Nevertheless, smaller incisions may lead to more pronounced early wound edema. While both incision sizes are effective, the 2.6 mm incision may offer better corneal endothelial protection and more stable fluidics in hard nuclear cataracts.

## Data Availability

The raw data supporting the conclusions of this article will be made available by the authors, without undue reservation.
